# Hard-template-engaged formation of Co_2_V_2_O_7_ hollow prisms for lithium ion batteries†

**DOI:** 10.1039/c7ra11373k

**Published:** 2018-01-09

**Authors:** Xuefeng Chu, Huan Wang, Yaodan Chi, Chao Wang, Lei Lei, Wentong Zhang, Xiaotian Yang

**Affiliations:** Jilin Provincial Key Laboratory of Architectural Electricity & Comprehensive Energy Saving, School of Electrical and Electronic Information Engineering, Jilin Jianzhu University Changchun 130118 China hanyxt@163.com

## Abstract

Highly uniform and monodispersed Co_2_V_2_O_7_ hollow nanoprisms are successfully fabricated *via* a simple hard-template-engaged strategy. In such synthesis, Co acetate hydroxide plays the important role of not only the cobalt source, but also the sacrificial template. After coating with a carbon layer, the as-obtained Co_2_V_2_O_7_ hollow nanoprisms exhibit apparently enhanced lithium-ion-battery performances. It has shown a high specific capacity of around 946 mA h g^−1^ at a current density of 100 mA g^−1^, excellent cycling stability up to 300 cycles at a current density of 1 A g^−1^, and superior rate performance, which is much better than carbon coated solid Co_2_V_2_O_7_ samples.

## Abstract 2

A novel hard-template-assisted method is developed to fabricate high quality Co_2_V_2_O_7_ hollow nanoprisms. When evaluated as anode materials for lithium ion batteries, they exhibited a remarkable enhanced performance compared with the solid ones.

## Graphical abstract

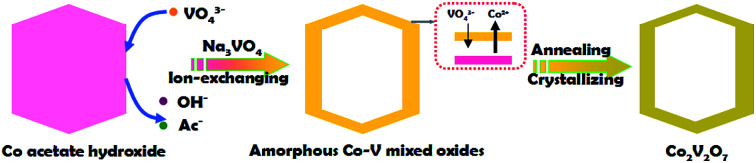

In the past decades, research on clean and environment-friendly energy resources, which are considered as the most efficient way to solve the increasingly serious energy crisis, has received continuous attention.^1–3^ Among the varieties of solutions, lithium ion batteries (LIB) are some of the most important energy storage devices due to their various merits of high voltage, high recharge capability, low cost, and environmental friendliness.^4–7^ However, there are still several challenging issues in the actual research and application, such as the low power density, relatively low capacity compared with the theoretical value as well as the problems of longer cycle life and better rate performance. Thus, developing new kinds of LIB materials, including component controlling, shape engineering and structure designing, is still the most important and urgent target to be achieved.^8,9^

Previous reports have firmly confirmed that inorganic nanoparticles (NPs) with hollow inter structures always exhibit highly increased LIBs performances compared with the solid ones,^10–15^ owning to their larger specific surface area, good accessibility, shorter distance over which lithium ions must diffuse in the solid state, and more importantly, the larger increased structural stability during the charge–discharge process. Among the variety kinds of available methods to prepare hollow structured materials, hard-template engaged strategy has become the research hot-spot very recently. Such synthesis involves the use of various active or inactive particles to support the deposition/growth of shell materials, followed by etching treatment to remove the template components. Compared with the template-free method, hard-template method has some important advantages: (1) the usage of hard template is more suitable to realize the precise timing of inner hollow space, fine structure of outer shell and even the whole morphology; (2) uniform hollow space is much easier to be fabricated; (3) it is an efficient way to couple multiple components together, that increasing the complexity of hollow structures in a designed manner may bring more possibilities in modulating the properties of functional nanostructures for many applications. In generally speaking, an idea hard template should have good activity with designed shell component and also be easily removed after the synthesis. With the rapid development of synthetic nanotechnology, many kinds of hollow NPs have been successfully prepared.^16–33^ For examples, using Cu_2_O sphere, cubic or octahedral particles as sacrificial templates, a series of metal oxide-based spherical or non-spherical nanocages can be prepared;^23–26^ Porous carbon or carbon/metal polyhedrons have been synthesized by directly annealing metal–organic-frameworks (MOFs) as hard templates.^16–22^ Otherwise, MnCO_4_ nanocubes,^27^ Cu nanowires,^28^ ZnSnO_3_ nanocubes,^29^ graphene nanosheets^30^ and *et al.* are also used as the hard templates to construct functional hollow nanomaterials. Very recently, a new kind of hard template, metal-Ac has been successfully developed to assist the formation of hollow NPs. As reported by Lou's group, metal-Ac is unstable. The precipitation can self-decomposed completely in aqueous solution, indicating the high activity. By utilizing such structural feature, a series of Co-based mixed metal oxides and sulfides are constructed,^31–33^ with excellent performances in energy-related areas.

Cobalt-based mixed metal oxides with different metal cations have demonstrated high electrochemical activities due to their interfacial effects and the synergic effects of the multiple metal species. For instance, cobalt vanadate is one of the most important materials for energy storage.^34–37^ That is because introducing vanadium to couple with cobalt to form binary cobalt vanadates can largely enhance the electronic/ionic conductivity, reversible capacity and mechanical stability compared with single-component cobalt oxides. Many kinds of cobalt vanadate NPs with tunable components and shapes been reported, including self-assembled Co_3_V_2_O_8_ nanosheets,^34^ Co_3_V_2_O_8_ sponge networks^35^ and Co_2_V_2_O_7_–graphene hybrid nanocomposites.^36^ However, to the best of our knowledge, hollow cobalt vanadates is rarely reported, except the current reported Co_3_V_2_O_8_·*n*H_2_O hollow hexagonal prismatic pencils with micron in diameters by Yu and co-workers synthesized *via* a hydrothermal approach.^37^ The challenges still exist in decreasing the particle sizes of cobalt vanadate and controlling the interior structures, as well as the compositions. Thus, it would be of great interest to develop a facile approach to synthesize novel cobalt vanadate with controllable hollow nanostructure.

Inspired by the previous reports, herein, we present a facile template-engaged approach for the synthesis of Co_2_V_2_O_7_ hollow prisms with controllable morphology and inner nanostructure *via* an ion-replacement reaction between Co acetate hydroxide and Na_3_VO_4_. Co acetate hydroxide plays the important role of not only the cobalt source, but also the sacrificed template. Impressively, after surface coating with thin carbon layer, the as-obtained hollow prisms manifest higher specific capacitance with enhanced cycling stability compared with the solid ones, making them potential electrode materials for LIBs.

The whole synthesis is summarized in the Scheme 1 (details are shown in the ESI†). In the first step, Co acetate hydroxide precursors were prepared as described by Lou's group.^31,32^ The morphology of the precursor is revealed by TEM as depicted in Fig. S1.† The result matches well with the previous report, that the precursors have uniform contrast, clearly indicating their solid and dense nature. The as-obtained 1-D nanoprisms are around 450 nm in length and 75 nm in width. Fig. S2† is the XRD data, which could be assigned to a tetragonal Co_5_(OH)_2_(CH_3_COO)_8_(H_2_O)_2_ phase.

**Scheme 1:**
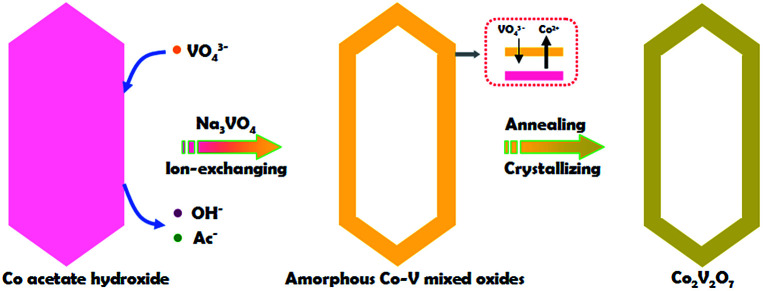
Schematic illustration of the synthesis of hollow Co_2_V_2_O_7_ sample.

Next, we chose Na_3_VO_4_ as the V source to reacted with Co acetate hydroxide owning to its high solubility in water. Generally, V-based raw materials always have poor aqueous solubility. For example, NH_3_VO_3_ is a common one, but it is hard to be dissolved in water unless excess H^+^ or OH^−^ ions are existed. However, the presence of free H^+^ or OH^−^ ions can strongly accelerate the dissolution or hydrolysis of Co acetate hydroxide, which is fatal for the following ion-exchange reaction. In addition, there are another two key points in the synthetic steps to avoid the self-decomposition of Co acetate hydroxide precursors: first, it is necessary to use absolute ethanol to pre-disperse Co-based precursors; the next one is the fast injection of Na_3_VO_4_ aqueous solution into the reaction system. With the typical photos shown in Fig. S3,† after adding Na_3_VO_4_ aqueous solution, the original pink solution has become to ochre colour within 20 seconds, clearly indicating the successful formation of ion-exchange reaction between Co acetate hydroxide and Na_3_VO_4_. A series of characterizations have been used to obtain the structural information of the product. With the data displayed in Fig. 1, the corresponding TEM images have shown that the product well maintained the original 1D prism-like nanostructure with slightly shrunken length (around 400 nm) and expended width (around 100 nm). Compared with the original smooth surface, the surface of the as-formed nanoprisms kept well. Interestingly, well-defined inner cavities could be found in every nanoprism, which could be easily distinguished by the sharp contrast between the center and the edge. Furthermore, the HAADF-STEM-Mapping technique was also used for the analysis of the fine distribution of the Co and V elements in the nanostructures. As shown in the image presented in Fig. 1d–f, both of the two elements spread throughout the hollow nanoprisms uniformly. The atomic ratio of Co and V is 1 : 1.07, which can also be calculated from the EDX data (with the data shown in Fig. S4†). The XRD pattern of the as-obtained product was also tested and is shown in Fig. 1c. No clear diffraction peaks could be found, indicating the product is in the form of amorphous phase.

**Fig. 1:**
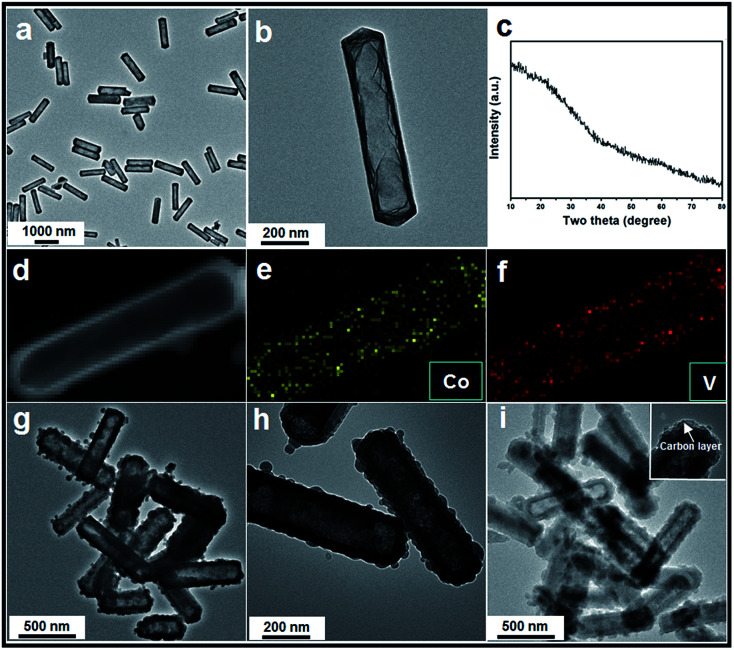
(a) and (b): TEM images of the as-obtained amorphous Co–V mixed metal oxides; (c) the corresponding XRD data; (d) to (f) HAADF-STEM-Mapping analysis of the Co–V mixed metal oxides; (g) and (h) TEM images of the Co_2_V_2_O_7_ obtained *via* a heating treatment; (i) TEM image of Co_2_V_2_O_7_@C core@shell composite.

A series of control experiments was designed to gain a deeper insight into the formation mechanism. Initially, we attempted to use water as solvent to instead of ethanol to disperse Co acetate hydroxide precursors. It is found that the as-obtained Co-based precursors are easily dissolved in water within a few minutes completely. Further using such clear solution to react with VO_4_^3−^ couldn't obtain ordered product, only irregular small sized nanoparticles are found from the TEM images shown in Fig. S5.† It could be understood that without the assistant of hard-template, the product is hard to keep uniform morphology. Next, we performed the synthesis under the same conditions, except at higher temperature (80 °C). Fig. S6† is the corresponding TEM result. The quality of the product is poor, and many broken areas are also found. But the hollow inner nanostructures are still well maintained. The possible reason could be attributed to the increased hydrolyzing trend of Co acetate hydroxide at higher temperature. Finally, we also investigate the effect of Na_3_VO_4_ feeding amount towards the final product by doubling the Na_3_VO_4_ amount. As shown in Fig. S7,† no obvious change could be found. The product is also high-quality and in the form of prism-like hollow nanostructure. Further ICP analysis is also been taken. The result confirmed that the molar ratio of Co and V is also around 1/1, indicating that such stoichiometric ratio is thermodynamic stable.

By referring to the above experiments, it is considered that the whole synthesis is a typical speed race process involving two independent reactions after addition of Na_3_VO_4_ aqueous solution: the hydrolysis of Co acetate hydroxide and the ion-replacement reaction between Co acetate hydroxide and Na_3_VO_4_. Unfortunately, we can not find the exact data about the solubility product constant (*K*_sp_) of Co acetate hydroxide as well as cobalt vanadate complex. But we noticed that Co acetate hydroxide can self-decompose in pure water but cobalt vanadate is stable enough, indicating the *K*_sp_ (cobalt vanadate) > *K*_sp_ (Co acetate hydroxide). Thus the ion-replacement reaction is driven by thermodynamics. Additional, our synthetic result has also shown that the binding energy between Co-(VO_4_) is much stronger than Co-(OH). As a result, Co–V mixed metal oxide is formed and Co–OH is allowable in thermodynamics. Further referring to the previous report, it is considered that a thin Co–V mixed metal oxide layer is *in situ* formed on the outer surface of Co acetate hydroxide precursor at the start of the reaction. Here, the as-generated Co–V mixed metal oxide acts as a physical barrier to hinder a direct chemical reaction between outside (VO_4_^3−^) ions and inner metal-ion species. Therefore, further reaction depends on the relative diffusion of metal or VO_4_^3−^ ions through this newly formed oxide layer. Along with the reaction going, the outward flow of Co^2+^ will consume the core template, leading to the generation of void space within the Co–V mixed metal oxide shells. Thus, the hollow nanostructure is formed.

Finally, the pre-synthesized amorphous Co–V mixed metal oxides are further annealed at 350 °C in air to accomplish the formation of crystallized phase. The corresponding XRD patterns are shown in Fig. S8,† the peaks between 2*θ* = 23.2° and 40.1° indicates the formation of well crystallized Co_2_V_2_O_7_ phase (JCPDS card no. 01-070-1189, space group *P*21/*n*). No additional diffraction peaks could be observed from the XRD patterns, suggesting the high purity of the products. From the low-magnification TEM image in Fig. 1g, it is clear the as-obtained products are uniform and monodisperse. The enlarged TEM image in Fig. 1h confirms the particle size and the inner hollow space of the annealed sample are kept well compared with the un-heated ones, indicating the unique structural stability of the hollow nanostructure. The only difference is the original smooth surface becomes much rougher. The thickness of the coated carbon layer is about 10 nm, which is obtained from the corresponding TEM images shown in Fig. 1i and inset. X-ray photoelectron spectroscopy (XPS) analysis is carried out to characterize the chemical state of the elements in Co_2_V_2_O_7_ hollow nanoprisms. As shown in Fig. S9,† the two main peaks at 786.5 eV and 803.0 eV that can be assigned to the Co 2p3/2 and 2p1/2 spin orbit peaks, respectively, whereas the peaks at 516.8 and 517.5 eV correspond well to the V 2p3/2 and 2p1/2 spin orbit, which firmly confirmed that the valence state of Co and V is +2 and +5, respectively. Additional, the Co and V contents were 35 and 33 wt%, respectively, as determined by elemental analysis using inductively coupled plasma atomic emission spectrometry (ICP-AES), that matches well with the EDX data of unheated sample. The specific surface area of the as-obtained Co_2_V_2_O_7_ hollow nanoprisms calculated from the BET curve (Fig. S10†) reaches approximately 97 m^2^ g^−1^. Such a large BET surface is possibly caused by the inner hollow structures.

The complexity of the hollow nanoprisms in both structure and composition might endow the materials with exceptional properties and performance in particular applications. In this work, we further demonstrate the remarkable lithium storage properties of the hollow Co_2_V_2_O_7_ nanoprisms, which are associated with their unique nanostructure and high complexity. Otherwise, solid Co_2_V_2_O_7_ nanoparticles and hollow Co_3_O_4_ nanoprisms are also synthesized and used as the references (details are shown in the ESI†). Before the LIB test, the three kinds of anode materials are further coated with carbon to further enhance (with the typical TEM images of carbon coated hollow Co_2_V_2_O_7_ nanoprisms shown in Fig. 1i and inset) their stabilities (experiment details and corresponding TEM images are shown in ESI†). Fig. S11† depicts the cyclic voltammogram (CV) profile obtained at a scan rate of 0.2 mV s^−1^ in the potential window of 3 V to 0.01 V. In the first cathodic sweep or during the lithiation step, two reduction peaks can be clearly identified at around 0.45, and 0.05 V, corresponds to the electrochemical reduction of Co_2_V_2_O_7_ to CoO accompanied by the lithiation of V_2_O_5_, further reduction into metallic Co and Li_1+*x*_VO_2_, respectively. Apparently, the peak intensity drops significantly in the second cycle, indicating the occurrence of some irreversible electrochemical processes in the first cycle. On the other hand, the anodic sweep of the first cycle shows two distinguished oxidation peaks at 1.29 and 2.36 V, which are ascribed to the formation of CoO from metallic Co and the delithiation of the vanadium oxides.

The discharge voltage profiles of the three carbon-coated samples at a current density of 100 mA g^−1^ are shown in Fig. 2a. For carbon coated Co_2_V_2_O_7_ nanoprisms, a distinct voltage plateau at *ca.* 1.21 V was clearly identified during the initial discharge process. The peak can be assigned to the transformation of Co_2_V_2_O_7_ into CoO accompanied by the formation of Li_*x*_V_2_O_5_ and the reduction of Co^2+^ to Co, which is in agreement with results reported in the literature.^34,37^ The rate capabilities of the three samples are investigated by gradually increasing the current density from 0.1 to 5 A g^−1^ and then returning it to 0.1 A g^−1^ (Fig. 2b). The average discharge capacities of the carbon coated Co_2_V_2_O_7_ hollow nanoprisms are 908, 876, 848, 732, 632 and 465 mA h g^−1^ at the current densities of 0.1, 0.2, 0.5, 1, 2 and 5 A g^−1^, respectively. Interestingly, when the current density was returned to 0.1 A g^−1^ after the 60 cycles, the specific capacity could be well recovered to 946 mA h g^−1^, which is even much better than the initiative ten cycles at 0.1 A g^−1^. For comparison, despite the carbon coated Co_3_O_4_ hollow nanoprisms and Co_2_V_2_O_7_ solid NPs also exhibit excellent rate and cycle capabilities, their specific capacities at all the current densities are much lower than carbon coated Co_2_V_2_O_7_ hollow nanoprisms. The values are 639, 588, 523, 472, 369, 267 mA h g^−1^ (for carbon coated Co_3_O_4_ hollow nanoprisms) and 770.8, 703.3, 647.5, 589.4, 547.6, 347.6, 766.3 mA h g^−1^ (for carbon coated Co_2_V_2_O_7_ solid NPs) at the current densities of 0.1, 0.2, 0.5, 1, 2 and 5 A g^−1^, respectively. The differences of the rate capacities between Co_2_V_2_O_7_ hollow nanoprisms and solid nanoparticles could be attributed to the shape-effect. Obviously, the presence of hollow nanostructure is more efficient than the solid one for LIB applications.

**Fig. 2:**
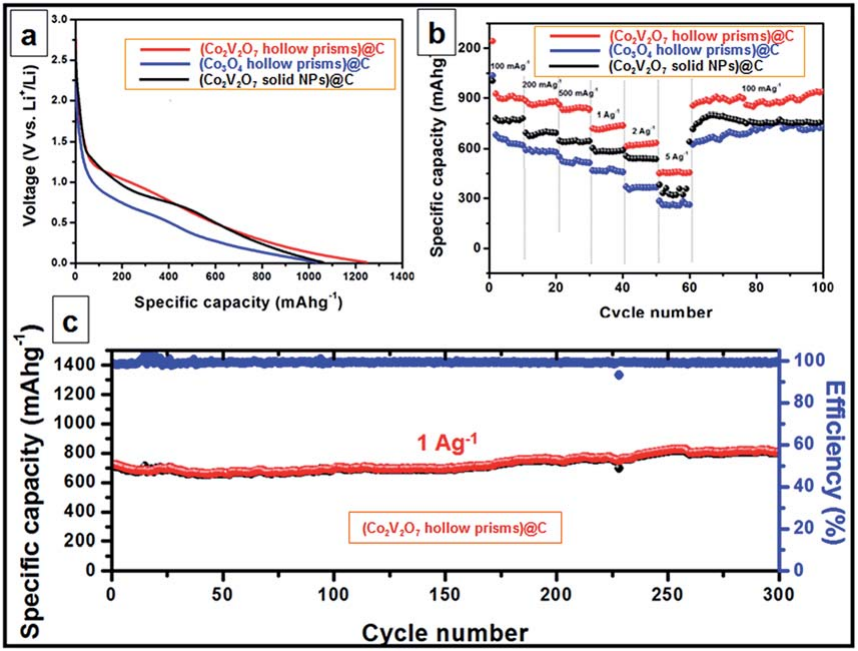
Electrochemical performance of carbon coated Co_2_V_2_O_7_ hollow nanoprisms (red line), carbon coated Co_3_O_4_ hollow nanoprisms (blue line) and Co_2_V_2_O_7_ solid NPs (black line). (a) Discharge voltage profiles of the three samples at a current of 100 mA g^−1^ in 1st cycle; (b) rate performance at different current densities; (c) cycling performance and corresponding CE of carbon coated Co_2_V_2_O_7_ hollow nanoprisms at a current of 1 A g^−1^.

The cycling performance of Co_2_V_2_O_7_ hollow nanoprism is depicted in Fig. 2C, at a constant current density of 1 A g^−1^ between 0.01 and 3 V. It is interesting to observe that the discharge capacity decreases slightly before the 20th cycle. Beyond that point, there is a slight increase in the discharge capacity until the 300th cycle. Finally, the specific capacity could reach to 806 mA h g^−1^. Such result is similar with the previously reported Co_3_V_2_O_8_·H_2_O sample.^37^ The capacity fading at the first 20 cycles could be attributed to the deconstruction of crystal Co–V–O mixed metal oxides, which makes the reduction of the conductivity. After early cycles, the electrolyte can gradually penetrate into the inner part of the active materials, thus the specific capacity is increased. For better comparison, the cycling performance of bare Co_2_V_2_O_7_ without the protection by carbon layer has also been tested. With the data shown in Fig. S12,† Under the identical test conditions, the bare Co_2_V_2_O_7_ hollow prisms exhibit much faster capacity fading, and a capacity of only around 393 mA h g^−1^ is retained after 100 cycles. The result indicates the important role of carbon layer to help to maintain the hollow structure and improve the electrical conductivity.

In all, highly uniform and monodisperse Co_2_V_2_O_7_ nanoprisms with ordered inner hollow space have been successfully fabricated *via* a facile hard-template-engaged method. It is found that the coupling reaction between Co^2+^ and VO_4_^3−^ has priority over the hydrolytic reaction of Co acetate hydroxide itself. Thus, the addition of Na_3_VO_4_ aqueous solution can cause an irreversible anion-exchanging reaction between Co acetate hydroxide and Na_3_VO_4_ driven by thermodynamics. After a facile annealing treatment in air, the amorphous Co–V–O mixed metal oxides can be transformed to crystal Co_2_V_2_O_7_ nanoprisms with a well-maintained hollow interior. The as-synthesized Co_2_V_2_O_7_ nanoprisms exhibit remarkable electrochemical performance as anode materials for high-performance LIBs by thin-carbon-layer coating. It is believed such “hard-template-assisted” strategy in the synthesis of mixed metal oxide with hollow interior is expected to be of great significance for the design and preparation of high efficient functional materials for clean energy.

## Conflicts of interest

There are no conflicts to declare.

## Supplementary Material

RA-008-C7RA11373K-s001
