# Supplementary material for: Hard-template-engaged formation of Co2V2O7 hollow prisms for lithium ion batteries
Source: RSC Adv. 2018 Jan 9;8(4):2072–6. doi: 10.1039/c7ra11373k (PMC9077232; doi:10.1039/c7ra11373k)
Supplement: RA-008-C7RA11373K-s001 [file RA-008-C7RA11373K-s001.pdf]

Electronic Supplementary Material (ESI) for RSC Advances.
This journal is © The Royal Society of Chemistry 2018

Hard-Template-Engaged Formation of $Co_2V_2O_7$ Hollow Prisms for Lithium Ion Batteries

Xuefeng Chu, Huan Wang, Yaodan Chi, Chao Wang, Lei Lei, Wentong Zhang, Xiaotian Yang*

Synthesis of Co acetate hydroxide precursors: The Co acetate hydroxide precursors are synthesized using the method reported by Lou's group.[31, 32] Typically, 0.68 g $Co(Ac)_2$ and 3 g PVP (K30) are dissolved in 200 mL ethanol at room temperature. Then the mixture is further heated at 85 ^oC in a oil bath for six hours. After cooling down to room temperature, the product is purified by centrifugation and washing with ethanol for three times. Finally the products are dried at 60 ^oC overnight.

Synthesis of Co-V-O hollow prisms: Typically, 40 mg Co acetate hydroxide precursor is dissolved in 40 mL ethanol using ultrasound for five minutes. At the same time, 0.2 mmol Na_3VO_4 is added in 10 mL H_2O to form a transparent solution. Then the Na_3VO_4 aqueous solution is injected into the ZIF-67 solution rapidly with continuously stirring. Two hours later, the products are collected by centrifugation and washed with ethanol for three times.

Annealing treatment: The above obtained precursors are further annealed in air at 350 ^oC for one hours (1 ^oC/min) to converse the precursors to mixed metal oxides.

Synthesis of solid $Co_2V_2O_7$: 0.2 mmol of $Co(NO_3)_2 \cdot 6H_2O$ and 0.4 mmol of Na_3VO_4 is dissolved in 40 mL of ethanol and 10 mL of H_2O, respectively. Then, the Na_3VO_4 aqueous solution is injected into the ZIF-67 solution rapidly with continuously stirring. Two hours later, the products are collected by centrifugation and washed with ethanol for three times. The as-obtained precursors are further heated in air at 350 ^oC for one hour.

Synthesis of hollow Co_3O_4 nanoprisms: The above obtained Co acetate hydroxide precursors are further annealed in air at 350 ^oC for one hours (1 ^oC/min) to converse the precursors to hollow Co_3O_4 nanoprisms.

Synthesis of carbon coated anode materials: The carbon coated samples are synthesized using the method reported by the previously literature (*Chem. Comm.* 2015, **51**, 6921). Typically, 50 mg of the synthesized product is dispersed in 30 mL of aqueous glucose solution (with 0.5 g of glucose) by ultrasonication. Then, the resulting suspension was transferred to a 50 mL of Teflon-lined autoclave, which was then heated at 170 ^oC for 8 h. the products are collected by centrifugation and washed with water for three times, followed dried at 80 ^oC over night. Finally, the products are annealed at 500 ^oC in N_2 for 2 hours at a heating rate of 1 ^oC/min to synthesize carbon coated anode materials.

Characterization: The X-ray diffraction patterns of the products were collected on a Rigaku-D/max 2500 V X-ray diffractometer with Cu-Kα radiation (λ = 1.5418 Å), with an operation voltage and current maintained at 40 kV and 40 mA. Transmission electron microscopic (TEM) images were obtained with a TECNAI G2 high-resolution transmission electron microscope operating at 200 kV. XPS measurement was performed on an ESCALAB-MKII 250 photoelectron spectrometer (VG Co.) with Al Kα X-ray radiation as the X-ray source for excitation.

LIBs test: The working electrode consists of 80 wt% of active material, 10 wt% of conductive carbon black, and 10 wt% of polymer binder (polyvinylidene fluoride, PVDF). The typical loading mass of the active materials is 1~2 mg cm^{-2} . The electrolyte is 1M $LiPF_6$ in a mixture of ethylene carbonate and diethyl carbonate (1:1 by weight). Lithium disc was used as both the counter and reference electrode. Cell assembly was carried out in an Ar-filled glovebox with moisture and oxygen concentrations below 1.0 ppm. The charge-discharge tests were performed on a LAND battery tester.

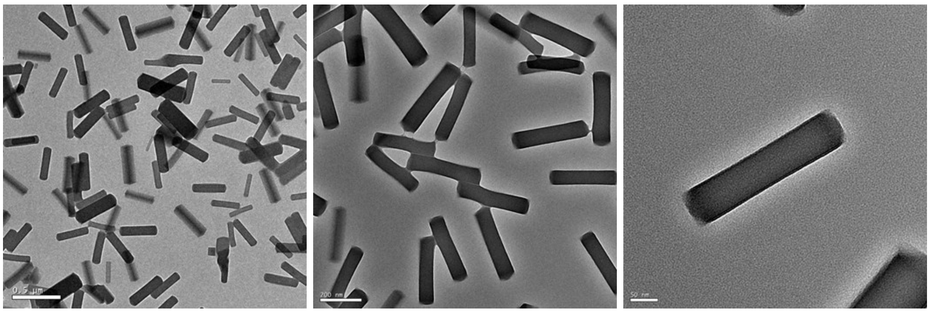

Figure S1. TEM images of Co-based precursors synthesized by Lou's reports.

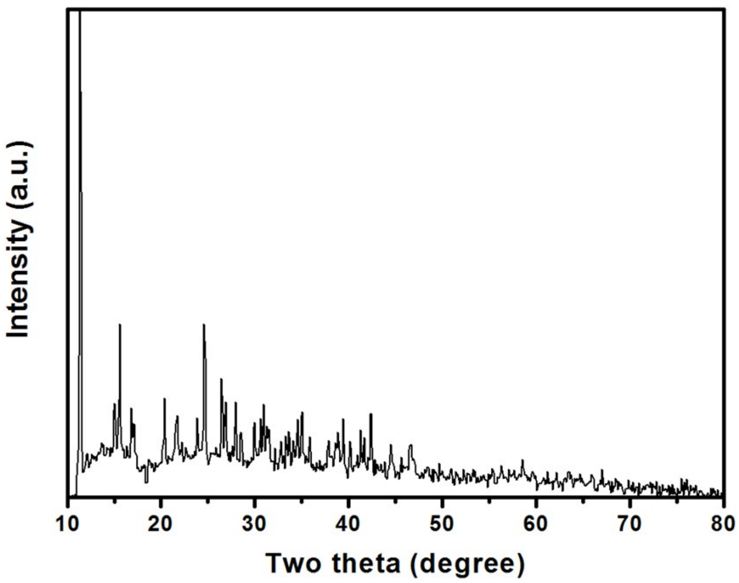

Figure S2. XRD data of the Co-based precursor.

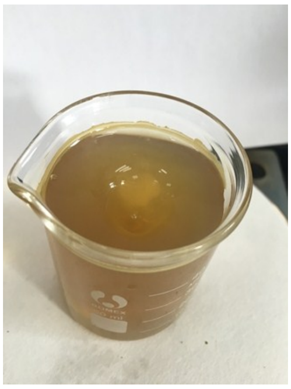

Figure S3. Photo of the reaction system which has been started for 20s after the addition of Na_3VO_4 aqueous solution.

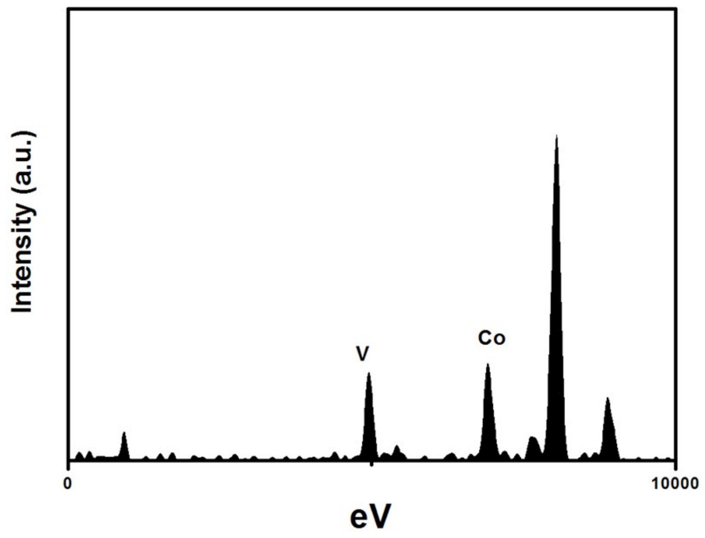

Figure S4. EDX data of the Co-V-O mixed metal oxide.

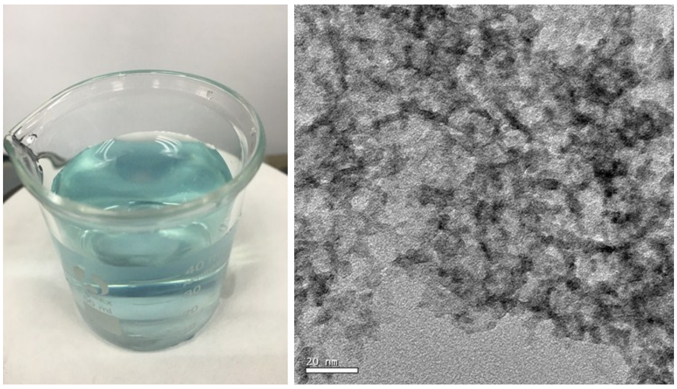

Figure S5. The photo (left) of the solution by mixing Co acetate hydroxide precursors and water together; the TEM image (right) of the product obtained by using such solution to further react with Na_3VO_4 aqueous solution.

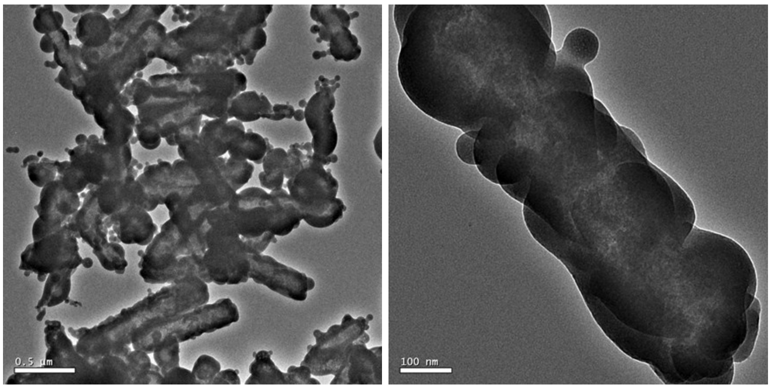

Figure S6. TEM image of the product obtained by increasing the reaction temperature to 80 ^{o}C.

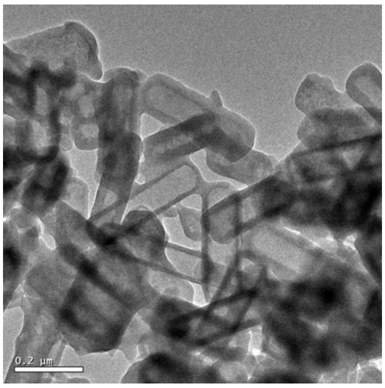

Figure S7. TEM image of the product obtained by doubling the Na_3VO_4 amount.

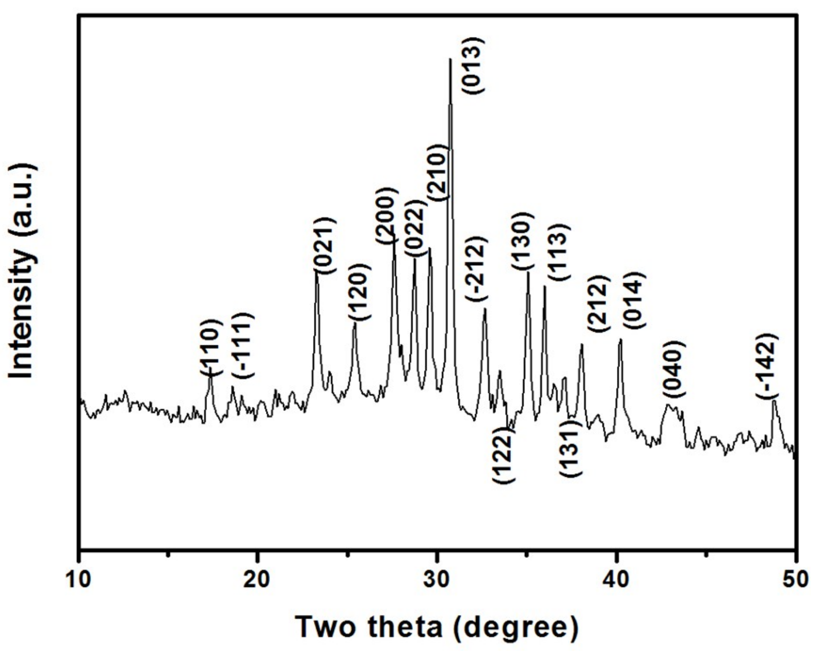

Figure S8. XRD data of $Co_2V_2O_7$ product.

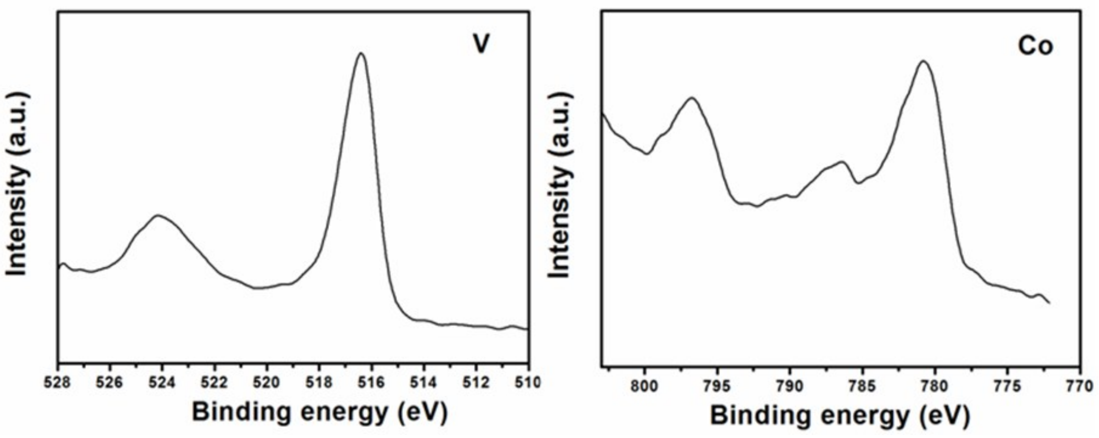

Figure S9. The XPS data of the hollow $Co_2V_2O_7$ sample after heating treatment.

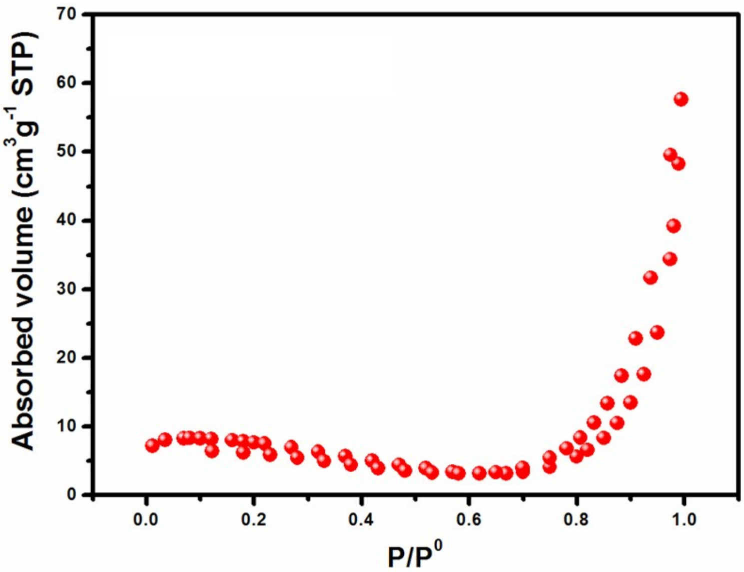

Figure S10. The BET curve of the annealed hollow $Co_2V_2O_7$ sample.

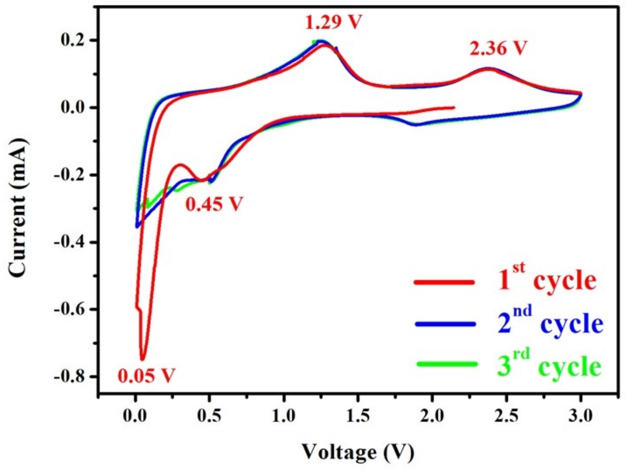

Figure S11. Cyclic voltammetry profile for the as-obtained carbon coated $Co_2V_2O_7$ hollow nanoprisms.

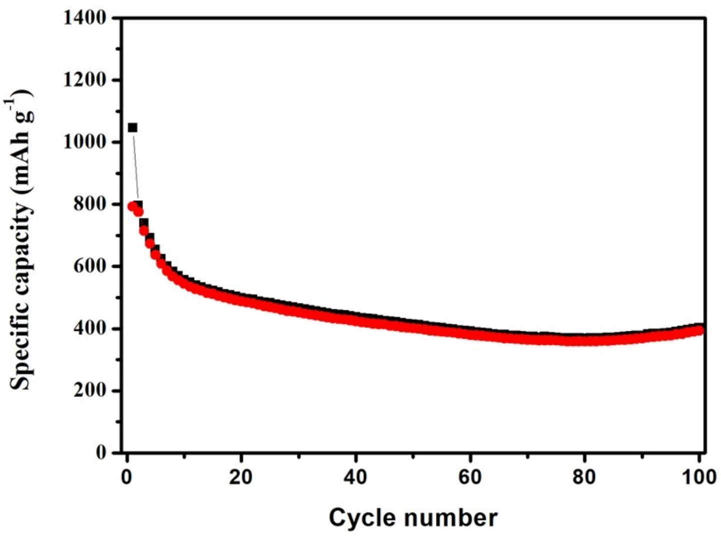

Figure S12. Cycling performance of bare $Co_2V_2O_7$ hollow nanoprisms without carbon coating at a current of 1A g^{-1}.
